# Disruption of transfer entropy and inter-hemispheric brain functional connectivity in patients with disorder of consciousness

**DOI:** 10.1186/1471-2202-14-S1-P83

**Published:** 2013-07-08

**Authors:** Verónica Mäki-Marttunen, Jesús M Cortes, Mirta F Villarreal, Dante R Chialvo

**Affiliations:** 1 Department of Cognitive Neurology, Institute for Neurological Research-FLENI, Buenos Aires, Argentina; 2 CONICET, Buenos Aires, Argentina; 3 Ikerbasque, The Basque Foundation for Science, E-48011, Bilbao, Spain; 4 Biocruces Health Research Institute. Hospital Universitario de Cruces. E-48903, Barakaldo, Spain

## 

Recent studies suggest that the functional organization of brain networks is altered in patients with severe disorders of consciousness (DOC), including coma [1]. A better characterization of these large-scale disturbances of brain function is fundamental for the understanding and eventual prediction of coma outcome. In this study we apply information theory tools and correlation measures to explore the brain functional connectivity (FC) in functional magnetic resonance imaging (fMRI) data from patients with DOC. In particular we are interested in the functional coherence within and between brain hemispheres as a measure of the integrity of brain function. The data analyzed comprises resting state fMRI data from 5 patients with traumatic DOC in two sessions, one early after the traumatic event and the second later in a stable clinical phase spanning several months. As a control we used similar data obtained from 17 healthy subjects. FC was assessed among time series of the blood oxygenated level dependent (BOLD) signal from 90 regions of interest (ROI) obtained using the Automated Anatomical Labelling (AAL) atlas [2]. These time series were used to estimate FC using two approaches: Partial Linear Correlation (PLC) and Transfer Entropy (TE) [3].

**Figure 1:**
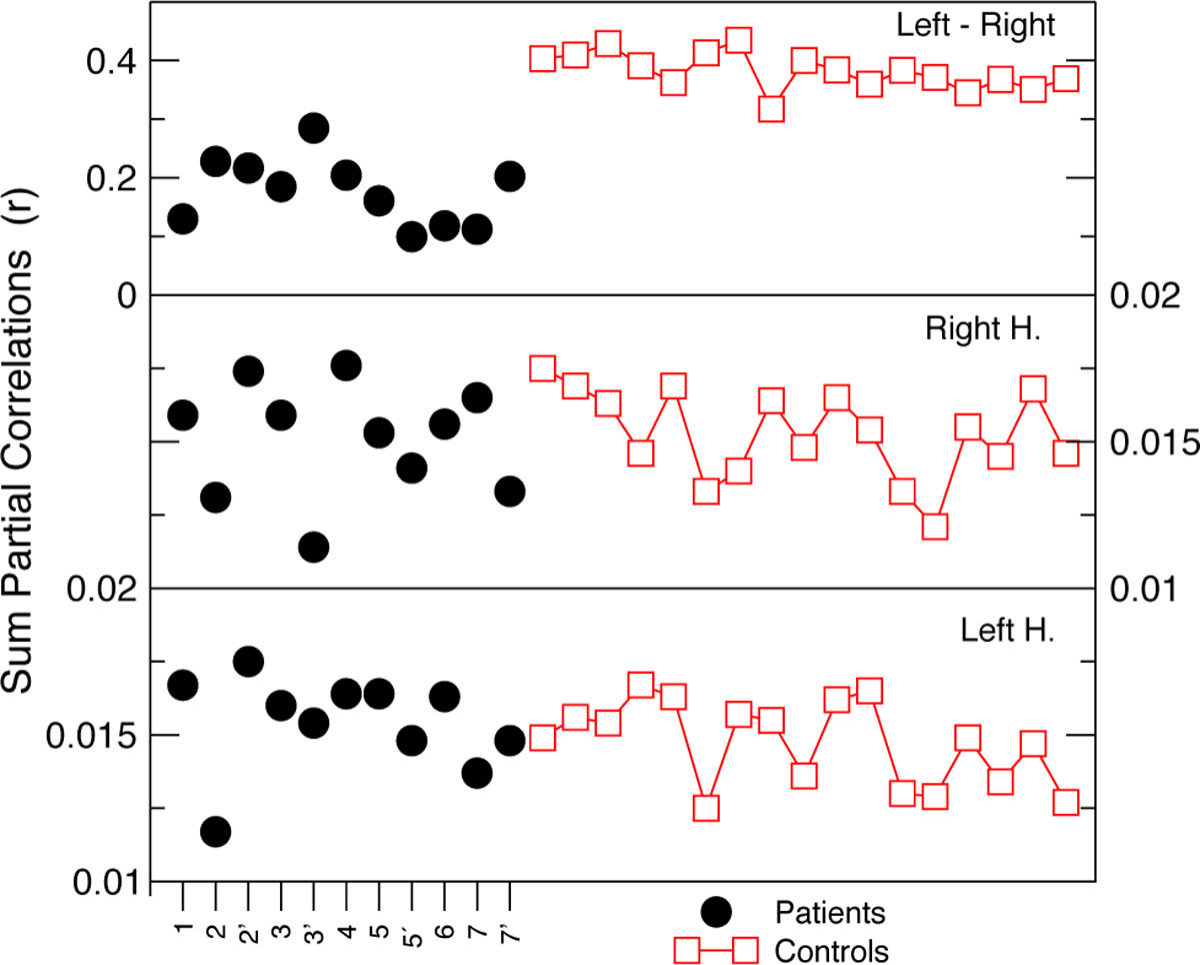
**For each subject it is shown the PLC summed over the 45 regions of the left hemisphere (bottom graph), the right hemisphere (middle graph) or between all right-left homologous ROIs in the AAL (top graph)**. Numbers on x-axis are indicating the different DOC patient; the quotes, the second fMRI sessions.

The major findings are summarized in Figure 1; the PLC analysis shows a significant reduction of the inter-hemispheric FC in DOC patients compared to controls. Regarding TE measures, we have found a strong reduction in the total TE of the DOC patients vs controls; mean values of 59.17 bits for DOC vs 123.97 bits for controls, whilst the two groups have a similar standard deviation (62.41 for DOC vs 76.69 for controls). Furthermore, the results based on TE seem to indicate that the inter-hemispheric FC asymmetry reported by the PLC in Figure 1 might be corresponded with an asymmetry between the TE travelling from "left to right" vs the one from "right to left" along the same ROI in the AAL. Notice that TE (but not PLC) is a non-symmetric measure. Interestingly, some of the DOC patients presented a FC pattern closer to the healthy subjects, for instance patient 3 who in the second session (i.e., 3' in Figure 1) was fully conscious. These findings might have clinical value for the diagnosis and design of recovery plans for patients with DOC.
